# No sex‐dependent mortality in an amphibian upon infection with the chytrid fungus, *Batrachochytrium dendrobatidis*

**DOI:** 10.1002/ece3.70219

**Published:** 2024-08-29

**Authors:** János Ujszegi, Nikolett Ujhegyi, Emese Balogh, Zsanett Mikó, Andrea Kásler, Attila Hettyey, Veronika Bókony

**Affiliations:** ^1^ Department of Evolutionary Ecology, HUN‐REN Centre for Agricultural Research Plant Protection Institute Budapest Hungary; ^2^ Department of Systematic Zoology and Ecology ELTE Eötvös Loránd University Budapest Hungary; ^3^ Department of Zoology University of Veterinary Medicine Budapest Budapest Hungary; ^4^ Doctoral School of Biology, Institute of Biology ELTE Eötvös Loránd University Budapest Hungary

**Keywords:** Bufonidae, Chytridiomycota, sex determination, sex‐specific survival, sexual development

## Abstract

One of the major factors driving the currently ongoing biodiversity crisis is the anthropogenic spread of infectious diseases. Diseases can have conspicuous consequences, such as mass mortality events, but may also exert covert but similarly severe effects, such as sex ratio distortion via sex‐biased mortality. Chytridiomycosis, caused by the fungal pathogen *Batrachochytrium dendrobatidis* (Bd) is among the most important threats to amphibian biodiversity. Yet, whether Bd infection can skew sex ratios in amphibians is currently unknown, although such a hidden effect may cause the already dwindling amphibian populations to collapse. To investigate this possibility, we collected common toad (*Bufo bufo*) tadpoles from a natural habitat in Hungary and continuously treated them until metamorphosis with sterile Bd culture medium (control), or a liquid culture of a Hungarian or a Spanish Bd isolate. Bd prevalence was high in animals that died during the experiment but was almost zero in individuals that survived until the end of the experiment. Both Bd treatments significantly reduced survival after metamorphosis, but we did not observe sex‐dependent mortality in either treatment. However, a small number of genotypically female individuals developed male phenotype (testes) in the Spanish Bd isolate treatment. Therefore, future research is needed to ascertain if larval Bd infection can affect sex ratio in common toads through female‐to‐male sex reversal.

## Graphical abstract

This paper is the first to examine whether chytrid infection causes sex‐dependent mortality in amphibians, which question is highly relevant for understanding host–pathogen interactions related to chytridiomycosis. We found that larval infection increased mortality after metamorphosis in males and females similarly. Furthermore, we also found a pattern suggesting long‐term negative effect of Bd infection on sex ratio via sex reversal of amphibians. This topic is highly important for the conservation of amphibians and should be further investigated, since such an unnoticed damage for the already dwindling amphibian populations may cause population extinction in some decades.

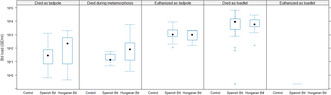

## 1 INTRODUCTION

The sixth mass extinction is happening in our present days (Ceballos et al., 2015). One of the major factors responsible for this biodiversity crisis is the spread of infectious diseases in wildlife populations, both directly and indirectly facilitated by anthropogenic activities and human‐induced environmental changes (Lindahl & Grace, 2015; Smith et al., 2009). Though amphibians did not suffer especially high losses in earlier extinction events, in the last century they became the most vulnerable class among vertebrates: more than 40% of amphibian species are threatened by extinction (IUCN, 2023; Luedtke et al., 2023). The globally spreading infectious diseases are among the main drivers of amphibian declines (Luedtke et al., 2023). Chytridiomycosis caused by the chytrid fungus *Batrachochytrium dendrobatidis* (Bd) has already led to the decline or extinction of several hundred amphibian species on all continents apart from Antarctica, due to repeated introductions arising from human activities (Lips, 2016; O'Hanlon et al., 2018; Scheele et al., 2019). The waterborne motile zoospores of Bd infect the keratinous epidermal layers of the amphibian skin (Berger et al., 1998). In case of serious infection in metamorphosed anurans, the structural damage caused in the skin can impair its osmoregulatory function leading to shifts in blood electrolyte balance. This may ultimately result in cardiac asystolic death (Voyles et al., 2009). Tadpoles exhibit keratinous elements only in their mouthparts (Marantelli et al., 2004), so they are less susceptible to Bd infection than later life stages, usually acting as reservoirs in natural habitats (Kilpatrick et al., 2010; Walker et al., 2010). However, even in tadpoles, sublethal Bd infection can negatively affect life‐history traits such as body mass, growth, and development (Blaustein et al., 2005; Garner et al., 2009; Hanlon et al., 2015; Parris & Cornelius, 2004), it may reduce the amount of skin‐secreted chemical defenses (Ujszegi et al., 2021) and often causes elevated levels of glucocorticoid “stress hormones” (Gabor et al., 2013, 2015, 2017). These sublethal effects can also contribute to increased mortality when combined with other stressors (McCoy & Peralta, 2018; Rohr et al., 2013).

In humans and other mammals, both genotypic (e.g., sex‐chromosome linked) and phenotypic (e.g., sex‐hormone dependent) differences between males and females mediate differences in the functioning of the immune system (Shannon et al., 2024); thus, parasites and pathogens often have greater impact on survival in one sex than in the other (Moore, 2003; Moore & Wilson, 2002). Such sex‐biased mortality can distort the sex ratio at the population level (Székely et al., 2014), and this can exacerbate the negative effects of infectious diseases on population viability (Rosa et al., 2019). Skewed sex ratios of breeding populations constrain effective population sizes and adaptive potential and can have cascading effects on other species and even ecosystems (Edmands, 2021; Mitchell & Janzen, 2010), although they may also facilitate the evolution of sex‐specific life histories and social systems (Schacht et al., 2022). However, sex differences in infection‐related mortality have been very little explored in wildlife epidemiological studies, especially in amphibians (Rosa et al., 2019), partly because identifying sex is not possible in most amphibians until they reach sexual maturity (Ujhegyi & Bókony, 2020), except for a few species where molecular sexing methods are available (reviewed by Nemesházi & Bókony, 2022). Despite the impact of and attention to Bd, no study, to our knowledge, has tested whether this pathogen causes differential mortality in male and female amphibians.

In this study, we experimentally investigated the lethal effects of larval Bd infection for several months after metamorphosis in common toads (*Bufo bufo*), and compared these effects between males and females using our recently developed molecular sexing markers for common toads (Nemesházi et al., 2022). This species is widespread and abundant across Eurasia (IUCN, 2023). Toad tadpoles can be experimentally infected with Bd, and costs of Bd infection can reach measurable levels during early life stages (Garner et al., 2009, 2011; Ujszegi et al., 2021; Woodhams et al., 2012), while adults tolerate Bd presence well with low infection intensities (Baláž et al., 2014; Vörös et al., 2018). The studied early life stages are especially relevant to our objectives because it is often in the aquatic larval stage when individuals become exposed to the waterborne Bd zoospores, while severe mortality usually arises upon metamorphosis, when Bd spreads all over the newly keratinized skin surfaces (Garner et al., 2009; Marantelli et al., 2004).

## 2 MATERIALS AND METHODS

### 2.1 Experimental procedures

All experimental procedures were approved by the Ethical Commission of the Plant Protection Institute, and permissions were issued by the Government Agency of Pest County (PE‐06/KTF/00754–8/2022, PE‐06/KTF/00754–9/2022, PE‐06/KTF/00754–10/2022, PE/EA/295–7/2018). The experiments were carried out according to recommendations of the EC Directive 86/609/EEC for animal experiments (http://europa.eu.int/scadplus/leg/en/s23000.htm).

In May 2022, we collected ca. 400 young tadpoles in development stages 26–28 (Gosner, 1960) using dip nets from a pond near Budapest, Hungary (Békás‐tó: 47.57638° N, 18.86918° E). It would have been preferable and we attempted to use individuals brought to the lab as embryos, but we could not do this because embryo mortality was unusually high both in the field and in the lab, presumably due to recurring bouts of sub‐zero temperatures, causing the pond to freeze over and damaging embryos at the collection site. We transported the tadpoles to the Experimental Station of the Plant Protection Institute in Julianna‐major, Budapest, and initially held them in groups of 38–40 tadpoles in transparent plastic containers (27 × 18 × 14 cm) in 5‐L reconstituted soft water (RSW; USEPA, 2002). During the first week, we maintained 16.3 ± 0.3°C (mean ± SD) temperature and set the photoperiod to match outdoor conditions. We fed the tadpoles with slightly boiled, chopped spinach twice a week. After 1 week, we selected 300 healthy‐looking tadpoles, and placed them in groups of 10 into 5 L RSW using the same type of containers. We picked the tadpoles at random (i.e., not by any randomization rule but without conscious choice, except that we did not include tadpoles that were too small or looked unhealthy). We released the remaining tadpoles back into their original habitat.

**FIGURE 1:**
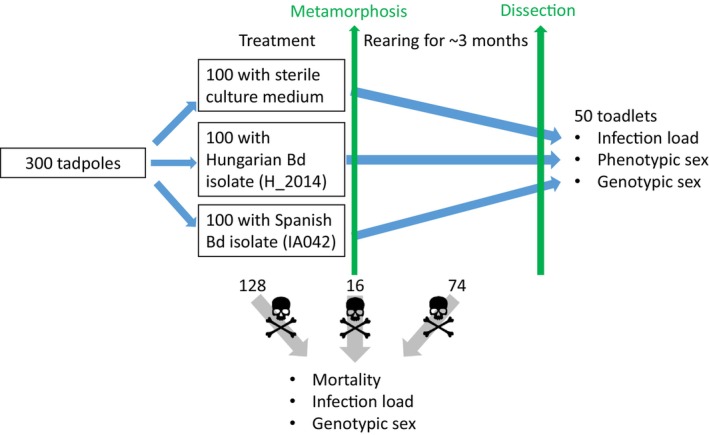
A schematic illustration of experimental treatments and processes exhibiting the number of treatment groups and replicates for each group. Skull and crossbones indicate individuals that died during the experiment.

From this point on, we exposed tadpoles during the entire larval development to sterile culture medium (control), or to liquid culture of one of two isolates of the global pandemic lineage (GPL) of Bd. The Spanish isolate (IA042) originated from a dead *Alytes obstetricans* collected in 2004 by T.W.J. Garner (Institute of Zoology, Zoological Society of London, UK) from a mass mortality event in the Spanish Pyrenees. The Hungarian isolate (Hung_2014) was collected from a living *Bombina variegata* in 2014 by J. Vörös (Department of Zoology, Hungarian National History Museum, Budapest, Hungary) in the Bakony Mountains, Hungary. We used these two isolates because they had been available in our laboratory; we did not aim to compare the effects of the two isolates since their culturing history is very different. We exposed individuals in groups of 10 to each of the three treatments in 10 replicates, resulting in a total of 30 experimental units and 300 tadpoles (Figure 1). We randomly assigned containers to the treatments and arranged the 30 rearing boxes into 10 spatial blocks, each containing one replicate from each treatment. Temperature was 18.4 ± 1.5°C (mean ± SD) during the experiment and we adjusted the photoperiod weekly to outdoor conditions. We fed tadpoles with spinach ad libitum and changed water twice a week using different dip nets for each treatment to prevent cross‐contamination. Three weeks after starting the treatments, we replaced the rearing containers with larger ones (31 × 20 × 16 cm) and reared each group of 10 tadpoles in 10 L RSW to halt an unexpected increase in mortality rates by providing a larger water volume for growing larvae. Bd exposure lasted until the start of metamorphosis for each individual (Figure 1).

When an individual reached development stage 42 (forelimb emergence), we weighed it to the nearest mg and placed it into a covered transparent plastic container (31 × 20 × 16 cm). We filled these containers with 250 mL RSW and lifted one side by ca. 2 cm to provide animals with both water and a dry surface. Once the animal reached development stage 46 (total tail resorption), we placed it into a transparent plastic container (21 × 16 × 12 cm) covered with a perforated lid, lined with wet paper towels and a piece of cardboard egg holder as a shelter. Each box contained five individuals originating from the same rearing box. After the completion of metamorphosis, we fed the toadlets ad libitum twice a week, initially with springtails (*Folsomia* sp.), then after 3 weeks with small crickets (*Acheta domestica*, instar stage 1–2) sprinkled with a 3:1 mixture of Reptiland (Art. Nr. 76,280; Trixie Heimtierbedarf GmbH & Co. KG, Tarp, Germany) and Promotor 43 (Laboratorios Calier S.A., Barcelona, Spain) to provide vitamins, minerals, and amino acids. We recorded the dates of starting metamorphosis, completion of tail resorption, and eventual mortality. Dead specimens were preserved in 96% ethanol. Ten weeks after starting the treatments, we euthanized tadpoles that had not started metamorphosis (*N* = 32) in a water bath containing 6.6 g/L tricaine methanesulfonate (MS‐222) buffered to neutral pH with the same amount of Na_2_HPO_4_, and preserved them in 96% ethanol.

To provide enough time to observe mortality patterns, we reared the individuals until 4 months after starting the treatments (Figure 1), by when the toadlets were between 7 and 14 weeks past metamorphosis. We euthanized all remaining individuals in a water bath using the same method as for non‐metamorphosed tadpoles. We removed both feet of each euthanized toadlet and stored them in 96% ethanol, while the rest of each toadlet's body was stored in 10% formalin.

### 2.2 Maintenance of Bd culture and exposure

Cultures were maintained in TGhL medium (mTGhL; 8‐g tryptone, 2‐g gelatine hydrolysate, and 4‐g lactose dissolved in 1‐L distilled water) in 25 cm^2^ cell culture flasks at 4°C and passed every 3 months into sterile mTGhL (OIE, 2016). One week before use, we inoculated 110 mL mTGhL with 3 mL of these cultures in 175 cm^2^ cell culture flasks and incubated these for 7 days at 21°C. We assessed the concentration of intact zoospores using a Bürker chamber at ×400 magnification and diluted both cultures to an equal concentration. After each water change, we inoculated 10 mL of these cultures into the tadpole‐rearing containers holding 10 L RSW, resulting in a mean initial concentration of ~1940 zoospores/mL in the rearing water. We inoculated control containers with the same quantity of sterile mTGhL. Contaminated water and equipment were disinfected overnight with VirkonS before disposal (Johnson et al., 2003).

### 2.3 Assessing Bd infection prevalence and intensity

To infer if the Bd treatment was successful, we analyzed 185 out of the 300 animals (Table 1; the remaining samples were unsuitable for Bd testing due to decay and one of them was accidentally lost). We assessed infection prevalence and intensity from dissected mouthparts of preserved tadpoles and from toe clips of metamorphosing and metamorphosed individuals. We homogenized tissue samples, and extracted DNA using PrepMan Ultra Sample Preparation Reagent (Thermo Fisher Scientific, Waltham, MA, USA) according to previous recommendations (Boyle et al., 2004), and stored extracted DNA at −20°C until further analysis. Following a standard amplification methodology targeting the ITS‐1/5.8S rDNA region (Boyle et al., 2004), we ran real‐time quantitative polymerase chain reactions (qPCR) on a BioRad CFX96 Touch Real‐Time PCR System (Bio‐Rad Laboratories, Hercules, CA, USA). To avoid PCR inhibition by ingredients of PrepMan, we diluted samples 10‐fold with double‐distilled water. We ran samples in duplicates, and in case of contradictory results, we repeated reactions in another duplicate. If these again returned an equivocal result, we considered that sample to be Bd positive (Kriger et al., 2006). Genomic equivalent (GE) values were estimated from standard curves based on five dilutions of a standard (1000, 100, 10, 1, and 0.1 zoospore genomic equivalents; provided by J. Bosch, Biodiversity Research Institute, University of Oviedo, Spain).

**TABLE 1:** Sample sizes and Bd prevalence (%) in each treatment group within each survival category.

Survival status	Treatment	N (total)	N (♂)	N (♀)	N (Bd)	Bd %
Died before metamorphosis	Control	48	26	22	8	0
	Spanish Bd	31^a^	15	15	15	93
	Hungarian Bd	49	20	29	19	89
Died during metamorphosis	Control	4	2	2	2	0
	Spanish Bd	5	4	1	5	100
	Hungarian Bd	7	5	2	7	100
Euthanized as tadpole (did not metamorphose)	Control	11	4	7	11	0
	Spanish Bd	10	3	7	10	100
	Hungarian Bd	11	8	3	10	100
Died after metamorphosis	Control	4	3	1	0	–^b^
	Spanish Bd	42	23	19	31	100
	Hungarian Bd	28	14	14	17	100
Euthanized as toadlet at the end of the experiment	Control	33	16	17	33	0
	Spanish Bd	12	3	9	12	8.3
	Hungarian Bd	5	2	3	5	0

Abbreviations: N (♂) and (♀), Number of males and females resulting from genotypical sexing; N (Bd), number of individuals analyzed for Bd.

^a^ Number of genotypically sexed individuals is 30 because one sample was lost from this group.

^b^ All samples in this group were unsuitable for Bd sampling due to rapid decay.

### 2.4 Sex determination

We extracted DNA from tissue samples using E.Z.N.A. Tissue DNA Kit following the manufacturer's protocol (except that digestion lasted at least 3 h). From animals that died prematurely as tadpoles or during metamorphosis, we used the tail, whereas from metamorphosed individuals we used one or two feet depending on size. For diagnosing genotypic sex, we used a DNA marker set published earlier (Nemesházi et al., 2022). For individuals that died before the end of the experiment, we used two markers (c16 and c2). These markers yielded the same result for each sample. For those individuals that survived to the end of the experiment, we used marker c16 only, and compared its result with the individual's phenotypic sex, because at the end of the experiment, the toadlets were old enough for their gonads to differentiate sufficiently (Ogielska & Kotusz, 2004). We categorized phenotypic sex as male (testes), or female (ovaries) based on the appearance of the gonads (before fixing in formalin) under an APOMIC SHD200 digital microscope. When phenotypic sex did not match the genotypic sex (marker c16), we used two additional markers (c2 and c5; both applied to two DNA samples isolated separately from each individual whenever it was possible), and in these cases, we also confirmed phenotypic sex by histological examination of the formalin‐stored gonads using routine histological procedures (Nemesházi et al., 2022).

### 2.5 Statistical analyses

All analyses were run in “R” (v4.3.2; R Core Team, 2023). For the analysis of survival, we used Cox's proportional hazards models (R package “survival,” function “coxph;” Therneau, 2023). We treated all euthanized individuals (i.e., those that survived until the end of the experiment, and those that were euthanized because they did not start metamorphosis) as censored observations. We measured survival in units of life stage (i.e., before, during, and after metamorphosis) rather than in days, to maximize sample size (because the exact date of death was lost for a few individuals) and to accommodate time‐dependent effects. Initial diagnostics suggested that the effect of Bd treatment was not constant over time, violating the proportional hazards assumption; therefore, we allowed for time‐dependent treatment effects by stratifying the model by life stage. First (model 1), we included only the interaction between treatment and life stage, and we estimated the differences between the control and Bd‐treated groups using the “emmeans” function of the “emmeans” package (Lenth, 2024), correcting the *p*‐values for false discovery rate (Benjamini & Yekutieli, 2001). To test whether mortality was sex‐dependent (model 2), we added the time‐independent effect of genotypic sex into the previous model. Then, to test whether the time‐dependent effect of treatment was sex‐dependent (model 3), we added the two‐way interaction between treatment and genotypic sex and compared this model with model 2 using a likelihood ratio (LR) test.

We did not analyze Bd prevalence and infection intensity statistically because the samples were taken whenever the individuals died or when we terminated the experiment, rather than at standardized time points for each treatment group. Because the deaths occurred scattered over 4 months between the start and the termination of the experiment, the comparability of infection intensity data is low, thus we used these data only to qualitatively assess if experimental infection with Bd was successful.

## 3 RESULTS

**FIGURE 2:**
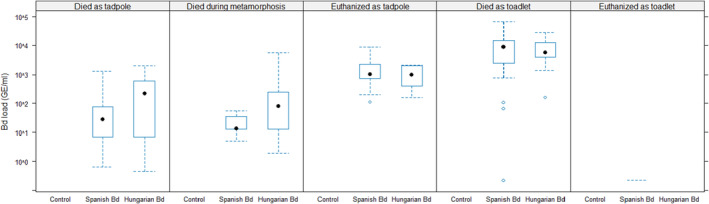
Infection intensity in Bd genomic equivalents (GE) in the three treatment groups within each survival category. In each box plot, the black dot is the median, the blue box is the interquartile range, and whiskers extend to the most extreme data points within 1.5 × interquartile range from the box; empty blue circles are data points >1.5 × interquartile range from the box. Note that only one toadlet euthanized at the end of the experiment had >0 Bd load (0.2 GE/mL), shown by a horizontal dashed line.

None of the control samples tested positive for Bd (Table 1). At the same time, experimental infection with Bd was successful, as infection with both isolates resulted in high prevalences and infection intensities in individuals that died before the end of the experiment (Table 1; Figure 2). However, out of the 17 Bd‐exposed individuals that survived until the end of the experiment, only one had a detectable Bd load (Table 1; Figure 2).

**FIGURE 3:**
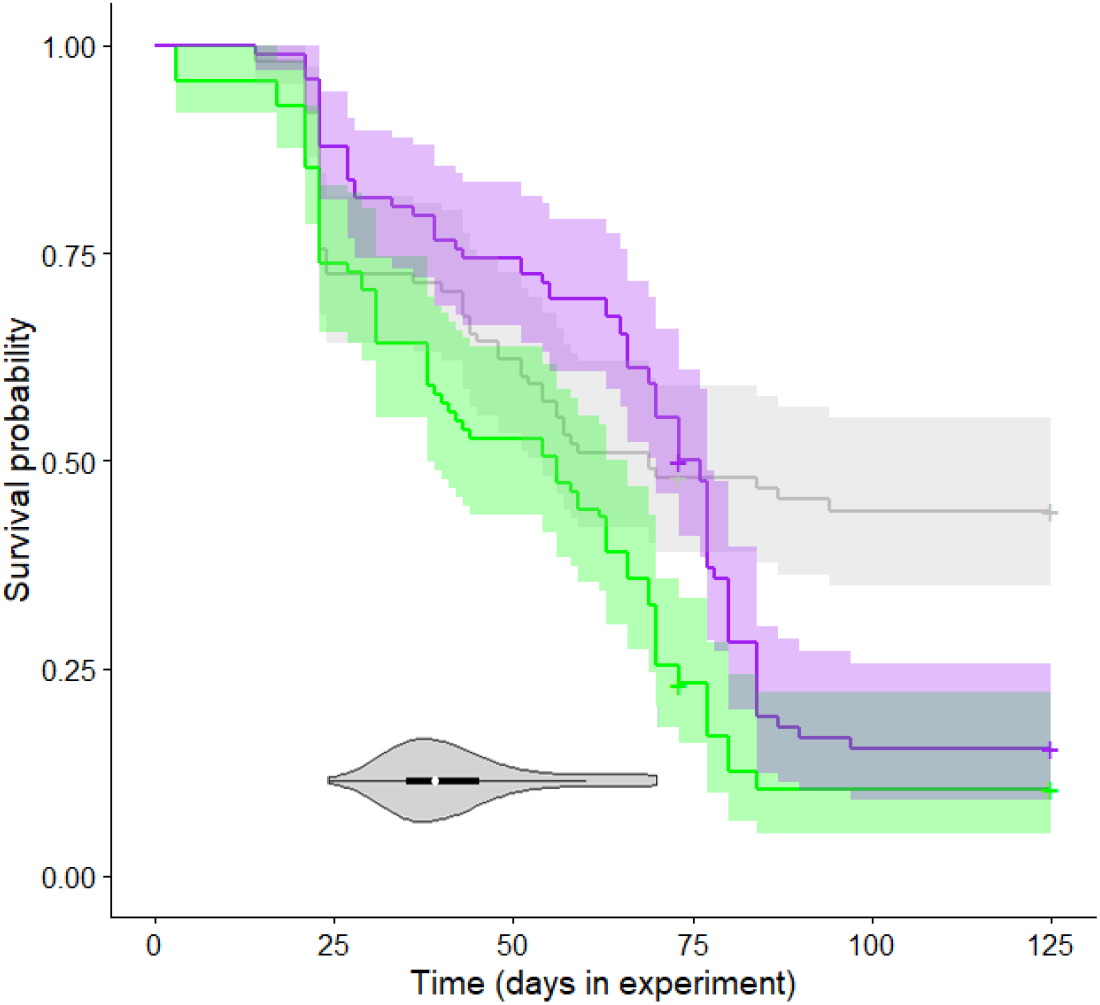
Survival over the experiment in the three treatments: control (gray), Hungarian Bd isolate (green), Spanish Bd isolate (purple), with 95% confidence intervals. The violin plot shows the starting dates of metamorphosis (white dot: median, black box: interquartile range, gray curved areas: Kernel density plots). Sample sizes are given in Table 1.

**FIGURE 4:**
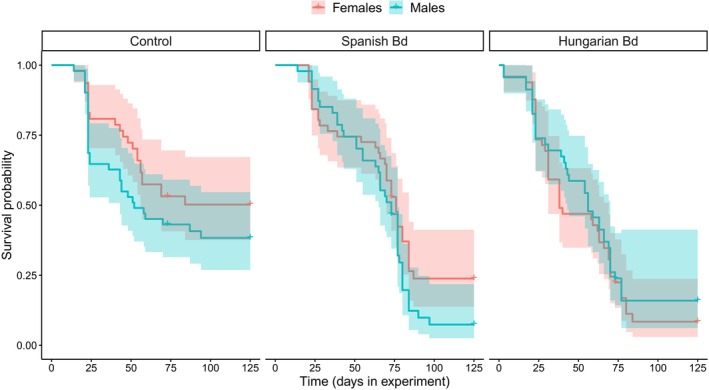
Survival over the experiment in the two sexes: genotypic females (red) and genotypic males (blue) with 95% confidence intervals separated by treatments. Sample sizes are given in Table 1.

Survival was low in the control group (Table 1), and it decreased further as a result of Bd treatment (Figure 3). Specifically, mortality after metamorphosis was significantly higher in the groups treated with the Hungarian or the Spanish isolate compared to the control group (Table 2), whereas there was no significant treatment effect during the tadpole stage and metamorphosis (Table 2). Genotypic sex did not affect survival either alone (model 2, female/male hazard ratio: 0.874 ± 0.119, *p* = .321; Table 1) or in interaction with Bd treatment (model 3, LR test: *χ* ^2^ = 3.012, df = 2, *p* = .222; Table 1; Figure 4).

**TABLE 2:** Effects of Bd treatments on mortality in three life stages, estimated from model 1.

Life stage	Comparison	Hazard ratio ± SE	*p*
Tadpole	Hungarian Bd/Control	1.028 ± 0.209	>.999
	Spanish Bd/Control	0.574 ± 0.132	.078
Metamorphosis	Hungarian Bd/Control	1.867 ± 1.170	>.999
	Spanish Bd/Control	0.863 ± 0.579	>.999
Toadlet	Hungarian Bd/Control	15.339 ± 8.247	<.001
	Spanish Bd/Control	12.886 ± 6.772	<.001

**FIGURE 5:**
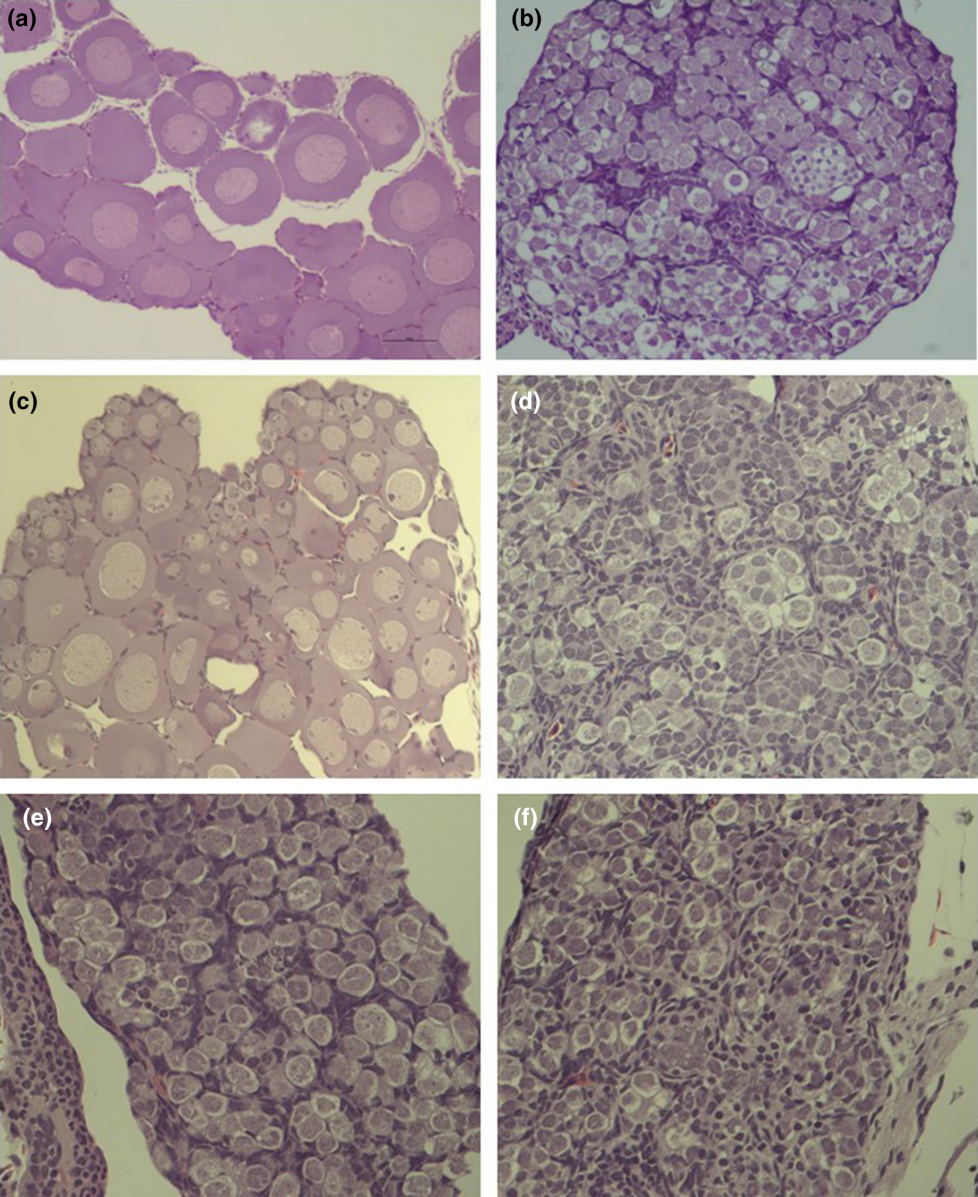
Histological images of toadlet gonads 7–14 weeks post‐metamorphosis. A sex‐concordant female (a) and male (b) are shown as reference from an earlier experiment (Nemesházi et al., 2022). In the present study, only the sex‐reversed individuals were examined histologically. The single genotypically male but phenotypically female individual (c) exhibited normal ovarian histomorphology with diplotene oocytes. The three genotypically female but phenotypically male individuals (d–f) exhibited normal testicular histomorphology with many somatic cells and seminiferous tubules full of gonocytes.

Surprisingly, in the group treated with the Spanish Bd isolate, three out of the nine genotypically female individuals that survived to the end of the experiment had male phenotype (Table 3). In these individuals, all three DNA markers corroborated female genotypic sex from two tissue samples of each individual, while both gonad morphology and histology corroborated male phenotypic sex (Figure 5). One of these three sex‐reversed individuals in the Spanish Bd treatment group was the only surviving toadlet that had a detectable Bd load at the end of the experiment. We did not find sex‐reversed individuals in the other treatment groups, except for a single toadlet in the control group that was genotypically male but phenotypically female (Table 3). In this individual, both genotypic and phenotypic sex were corroborated as above, except that we had only one tissue sample for genotypic sexing.

**TABLE 3:** Distribution of combinations of genotypic and phenotypic sex across the treatment groups.

Sex	Treatment group		
	Control	Spanish Bd	Hungarian Bd
Genotypic females			
Phenotypic females	17	6	3
Phenotypic males	0	3^a^	0
Genotypic males			
Phenotypic males	15	3	2
Phenotypic females	1^b^	0	0

^a^ Female‐to‐male sex reversal.

^b^ Male‐to‐female sex reversal.

## 4 DISCUSSION

Our data on Bd prevalence and infection intensity measured on individuals that died before the end of the study prove that our experimental exposure successfully infected larval toads. Interestingly, except for one individual with a very weak Bd load, juvenile toads that survived until the end of the experiment showed no detectable infection intensity. Whether these individuals did not become infected during larval development, or cleared infection after metamorphosis remains unknown. Successful infection also manifested in higher mortality of Bd‐exposed individuals compared to the control group, but only after metamorphosis, thus we found that the effect of Bd treatment on survival was time‐dependent. In the early weeks of the experiment, when mortality was most likely due to cold stress experienced during the embryonic stage and perhaps to the addition of the culture medium on which microbes grow very well, survival did not differ significantly across treatment groups. In contrast, after metamorphosis, animals that had been exposed to Bd during larval development showed significantly higher mortality. This agrees with previous findings that the survival of common toads after metamorphosis can sharply decrease upon infection with Bd (Bielby et al., 2015; Garner et al., 2009); however, in our earlier experiments, we did not experience a significant effect of Bd exposure with the same isolates on mortality of newly metamorphosed individuals originating from Hungarian common toad populations (Kásler et al., 2022; Ujszegi et al., 2021). This discrepancy may be explained by differences in the timing and/or duration of Bd exposure and the age of animals at Bd sampling. Furthermore, in the year of the present study, weather conditions were extremely unfavorable for amphibian breeding in the spring, due to drought and short warm periods followed by repeated episodes of harsh frost. Consequently, the wild‐caught tadpoles we used in the experiment had also been exposed to severe cold stress during their embryonic development. This probably contributed to the slow development and relatively high mortality in the control group (Beattie et al., 1991; Wersebe et al., 2019), and additionally to reduced Bd‐tolerance, since high temperature variability can increase amphibian susceptibility to Bd infection (Raffel et al., 2013). Therefore, the overall high mortality in the present study was likely due to multiple stressors, and not only to Bd exposure.

We found no effect of genotypic sex on survival, despite that mortality was relatively high throughout the experiment and, hence, would have allowed sex‐specific differences to manifest. Furthermore, the effect of Bd treatments did not differ significantly between males and females. These results suggest that early‐life mortality is not sex‐dependent in common toads, similarly to other species (Bókony et al., 2021), and the lethal effects of Bd infection are unlikely to skew sex ratios of young animals through sex‐biased mortality. Ranavirosis, the second most serious disease threatening amphibian biodiversity (Balseiro et al., 2009, 2010; Price et al., 2014) can alter sex ratio in Bosca's newt (*Lissotriton boscai*) breeding populations via increased mortality of females (Rosa et al., 2019). We are not aware of further studies that had investigated sex‐dependent mortality caused by an infectious disease in amphibians. However, sex differences in susceptibility to infections and mortality risk can vary across age groups (Klein & Flanagan, 2016), so it remains important to include both sexes and to investigate and report sex‐specific responses in studies of pathogen effects in various life stages of amphibians.

While examining the sex of the toadlets that survived until the end of the experiment, we detected the incidence of female‐to‐male sex reversal in some animals exposed to the Spanish Bd isolate (Table 3). We did not observe this effect with the Hungarian Bd isolate, which might be because the number of surviving toadlets was even smaller in this group than in those exposed to the Spanish isolate (Table 3). It is possible that the rates of sex reversal were higher than detected given the high mortality; however, we could not identify the phenotypic sex of individuals that died before dissection because it cannot be diagnosed reliably before sufficient gonad differentiation which occurs several months after metamorphosis in common toads (Ogielska & Kotusz, 2004). Nevertheless, the proportion of genotypic females that developed male phenotype (three out of nine toadlets in the Spanish Bd group) is remarkably high considering the following facts. First, the common toad seems relatively resistant to sex reversal, as in earlier studies we found only a single sex‐reversed individual among several hundreds of wild‐caught adults (Nemesházi et al., 2022), and no sex reversal in laboratory experiments where we exposed hundreds of tadpoles to potentially endocrine‐disrupting chemicals or heat treatments, except a high‐concentration chronic treatment with ethinylestradiol which caused male‐to‐female sex reversal (Bókony et al., 2020; Nemesházi et al., 2022; Ujszegi et al., 2022). Second, the direction of sex reversal we observed in the Spanish Bd treatment (i.e., female to male) is unexpected because the common toad has a ZZ/ZW sex chromosome system. According to the theory of asymmetrical sex reversal and scant empirical data, the homogametic sex is more prone to undergo sex reversal (Nemesházi & Bókony, 2022); thus, in common toads, ZZ females are more likely than ZW males. The single ZZ female we detected in the control group might be due to this higher propensity of sex reversal in the ZZ genotype, possibly in response to early‐life stressors experienced before we captured the tadpoles for our study. However, caution is warranted here, because we could not validate this single instance of male‐to‐female sex reversal with repeated genotypic sexing due to the missing of a second tissue sample from this individual.

Theoretical models predict that sex reversal has important implications for microevolution and population persistence, and the consequences depend on the type of the sex chromosome system and the direction of the sex‐reversing environmental effect (Bókony et al., 2017; Grossen et al., 2011; Nemesházi et al., 2021; Schwanz et al., 2020). For example, female‐to‐male sex reversal in ZZ/ZW species results in the production of WW offspring when sex‐reversed ZW males reproduce, which can either hasten extinction if the WW genotype is lethal, or it can catalyze an evolutionary transition to an XX/XY system and thereby save the population from extinction if the WW genotype is viable (Bókony et al., 2017; Nemesházi et al., 2021). Although there are no data on the viability of WW offspring in common toads, it might be inferred low based on the complete lack of such a genotype in free‐living toad populations (Nemesházi et al., 2022). Thus, if Bd infection causes female‐to‐male sex reversal, the production of ZW males (and thereby WW offspring) may have negative consequences for population persistence. Although the unexpectedly high mortality in our experiment constrains our sample size for investigating sex reversal, our findings highlight that further experiments are needed to elucidate if Bd infection causes sex reversal, in which species, and in which direction, as this information will be necessary for fully understanding the impact of Bd on biodiversity. Since the identification of sex reversal hinges on the availability of genotypic sexing methods (Nemesházi & Bókony, 2022), a sex‐reversing effect of Bd infection might be hidden in nature and could pose a serious threat to the already declining populations of several amphibian species.

Although it has been shown in several invertebrates that parasites and pathogens can influence sexual differentiation (Cordaux et al., 2004; Narita & Kageyama, 2008; Rodgers‐Gray et al., 2004), to our knowledge, this is the first documentation of sex reversal potentially triggered by a pathogen in vertebrates. Such an effect might be mediated by the elevated levels of glucocorticoid hormones that have been documented in many amphibian species in response to Bd infection (Gabor et al., 2013, 2015, 2017); the same hormones mediate environmentally induced sex reversal in various fishes although not in some others, nor in some amphibians and reptiles (Castelli et al., 2021; Goikoetxea et al., 2022; reviewed by Bókony et al., 2024). Whether Bd infection may cause sex reversal through the glucocorticoid stress response remains to be tested in future studies.

In conclusion, our results indicate that Bd infection is unlikely to disturb the sex ratio of common toad populations through sex‐specific mortality of infected tadpoles and young juveniles, but it might cause some genotypic females to develop into phenotypic males. This latter finding needs corroboration because of the small sample size of genotypic females that survived in our study, but it highlights that the possible relationship between sex reversal and Bd exposure deserves further attention in amphibians. Moreover, testing for sex‐dependent mortality effects of Bd in other species and of other pathogens will also be important for understanding and mitigating the drivers of population declines.

## AUTHOR CONTRIBUTIONS

**János Ujszegi:** Conceptualization (lead); data curation (equal); funding acquisition (equal); investigation (equal); methodology (equal); project administration (equal); writing – original draft (lead); writing – review and editing (equal). **Nikolett Ujhegyi:** Data curation (equal); investigation (equal); methodology (equal); project administration (equal); writing – review and editing (equal). **Emese Balogh:** Data curation (equal); funding acquisition (equal); investigation (equal); methodology (equal); writing – review and editing (equal). **Zsanett Mikó:** Investigation (equal); methodology (equal); project administration (equal); writing – review and editing (equal). **Andrea Kásler:** Data curation (equal); funding acquisition (equal); investigation (equal); methodology (equal); project administration (equal); writing – review and editing (equal). **Attila Hettyey:** Conceptualization (equal); funding acquisition (lead); investigation (equal); supervision (equal); writing – original draft (equal); writing – review and editing (equal). **Veronika Bókony:** Conceptualization (lead); data curation (equal); funding acquisition (lead); investigation (equal); methodology (equal); project administration (equal); supervision (equal); validation (equal); writing – original draft (equal); writing – review and editing (equal).

## CONFLICT OF INTEREST STATEMENT

The authors have no conflict of interest to declare.

## Data Availability

All data and script used in the analyses are available from Figshare Repository. DOI: 10.6084/m9.figshare.25118186.
